# Peculiar Structural Phase of a Single-Atom-Thick Layer of Antimony

**DOI:** 10.1021/acs.nanolett.3c02847

**Published:** 2023-10-20

**Authors:** Agnieszka Stȩpniak-Dybala, Tomasz Jaroch, Mariusz Krawiec, Piotr Dróżdż, Mariusz Gołȩbiowski, Ryszard Zdyb

**Affiliations:** Institute of Physics, Maria Curie-Sklodowska University, 20-031 Lublin, Poland

**Keywords:** 2D materials, antimonene, STM, LEEM, LEED, DFT

## Abstract

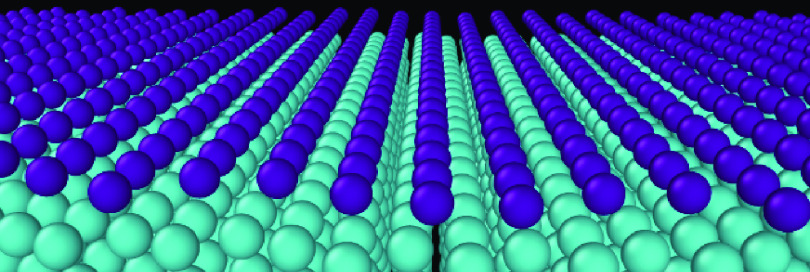

Using molecular beam epitaxy, a new structural phase of a single atom thick antimony layer has been synthesized on the W(110) surface. Scanning tunneling microscopy measurements reveal an atomically resolved structure with a perfectly flat surface and unusually large unit cell. The structure forms a well-ordered continuous film with a lateral size in the range of several millimeters, as revealed by low energy electron microscopy and diffraction experiments. The results of density functional theory calculations confirm the formation of a new phase of single-atom-thick antimony film without the buckling characteristic for the known phases of antimonene. The presented results demonstrate a substrate-tuned approach in the preparation of new structural phases of 2D materials.

Two-dimensional (2D) materials attract broad research interest due to their unique quantum phenomena and tunable functional properties. In a search for new single-atom-thick materials, theoretical models often consider free-standing films without any support, with an assumption of a negligible interaction between both subsystems. As a result, numerous structural phases of 2D materials are proposed, among which only some are feasible in practice. Antimonene is a good example of such a 2D system: several different crystallographic phases have been theoretically predicted,^1,2^ and only two of them, namely, α and β, have been experimentally realized.^3,4^ On the other hand, a substrate used in the bottom-up approach experiments can significantly modify thermodynamics and energetics of the formation of single-atom-thick materials. Obviously, the addition of a substrate can influence the symmetry and structure of the growing 2D materials. One of many examples of such 2D systems is silicene, which cannot be formed as a free-standing layer. Instead, depending on the substrate, it forms numerous phases with characteristic reconstructions,^5^ including an exceptional case of the planar form.^6,7^ Nevertheless, in all cases, the silicon atoms are arranged in the honeycomb lattice, as revealed by experiments and theory.

In the case of single-atom-thick films of antimony, besides the α and β phases of antimonene, there are very few reports on the successful formation of other ordered phases. Different approaches have been used to form Sb films or arrays of quasi one-dimensional structures on semiconductor substrates. In those cases, the Sb atoms are involved either in surface reconstruction or form a mixture of different materials.^8−11^ In the case of deposition on metals, most reports are devoted to Sb grown on noble metals. However, such systems are known from surface alloying, which occurs at low Sb coverages. In some cases, the appearance of alloy is followed by the growth of the known phases of antimonene.^12−14^ To our knowledge, there is one exception in which the successful formation of a single layer of antimony on AuSb alloy was realized. The reported rectangular unit cell resembles a double unit cell of one of the sublattices of the α-phase antimonene.^15^

Here, we report on the synthesis of a new structural phase of a single-atom-thick layer of antimony. The layer does not form an alloy with the underlying substrate; therefore, it is assured that no mixing of atoms between both elements occurs. Using low energy electron microscopy (LEEM), we demonstrate homogeneous and large scale growth of the layer. Its high quality and crystallographic order are confirmed by low energy electron diffraction (LEED) patterns. Atomically resolved scanning tunneling microscopy (STM) measurements together with the results of density functional theory (DFT) calculations show the perfect flatness of the layer without atom buckling. The arrangement of the Sb atoms in the layer reproduces the periodicity of the tungsten substrate along the [001] direction. Contrarily, it is incommensurate in the perpendicular direction, resulting in an unusually long unit cell and a periodicity of about 4.7 nm.

The results of STM measurements reveal that the imaged surface morphology strongly depends on a sample-tip bias, as shown in Figure 1. In the STM image presented in Figure 1a there are chains of atoms parallel to the W [001] direction. The distance between the atoms in the chains equals 3.1 ± 0.5 Å, which perfectly agrees with the distance between the W atoms along the [001] direction (3.165 Å). The average distance between the chains is 8.1 ± 0.5 Å, Figure 1b.

**Figure 1:**
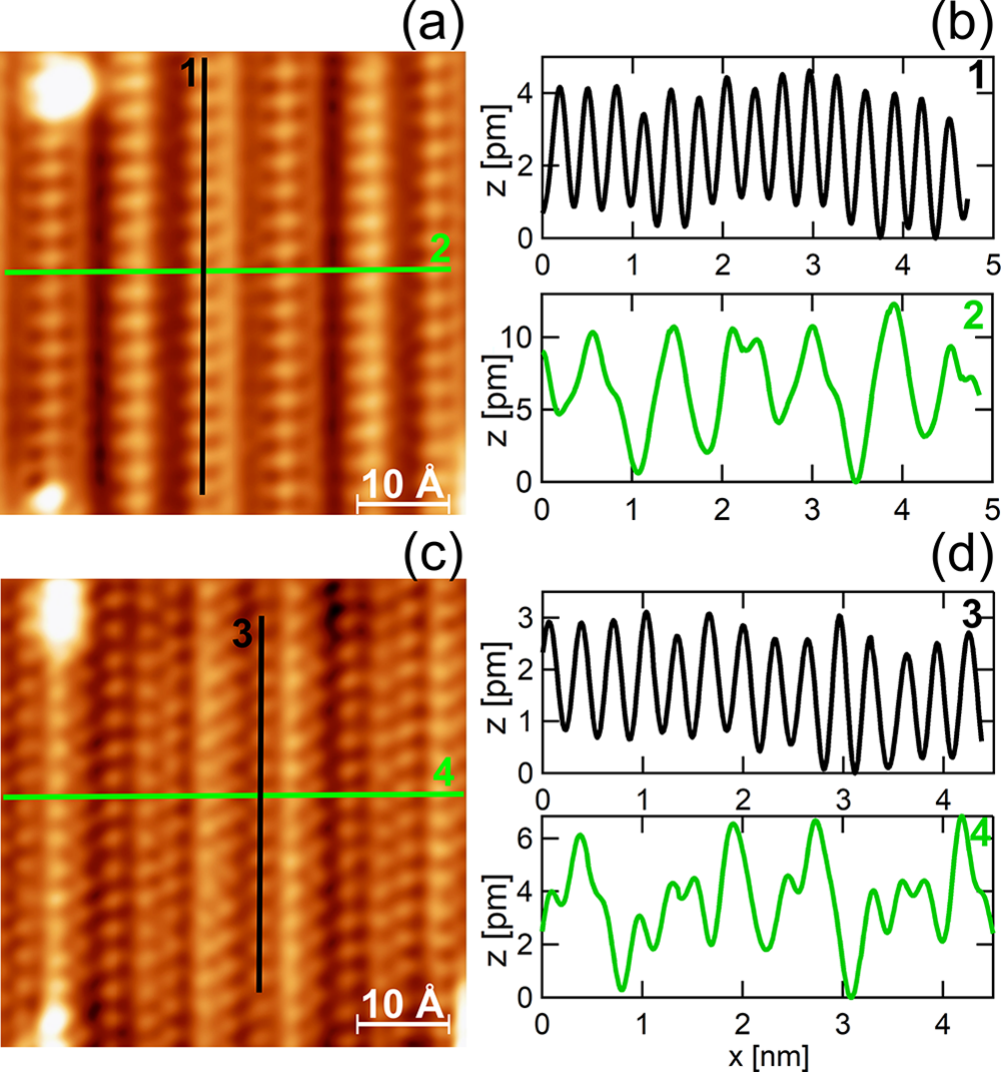
STM images (5 × 5 nm^2^) of the Sb monolayer on W(110) with corresponding profiles (b) and (d). (a) *U* = +1.0 V, *I* = 20 pA, (c) *U* = −1.2 V, *I* = 50 pA.

The Sb atoms that form the whole layer are perfectly resolved at negative sample polarization, Figure 1c. The layer is built of parallel atomic chains running along the W [001] direction. It is worth noting that the atom periodicity of 3.1 ± 0.5 Å along the chains is the same in each row of atoms. A characteristic feature of that image is in-phase arrangement of the Sb atoms in some of the neighboring chains and clear shift in the relative position in others. The distance between the individual chains changes from row to row between 2.5 and 3.6 Å, as shown in Figure 1d. The same arrangement of atoms in the neighboring chains, it means the length of the unit cell along the [11̅0] direction, is repeated every 15 rows, and it equals 47 ± 1 Å. Such unusually long periodicity is exceptional in the world of two-dimensional materials.

The LEED measurements support the STM observation on a larger scale. After the completion of the Sb monolayer the LEED patterns show a set of new sharp diffraction spots in addition to those characteristic to the bare W(110) surface, Figure 2. The new spots are located between the integer spots of the tungsten substrate along the [11̅0] direction, Figure 2b. It means that the new layer of Sb atoms has the same lattice constant as the tungsten substrate along the [001] direction.

**Figure 2:**
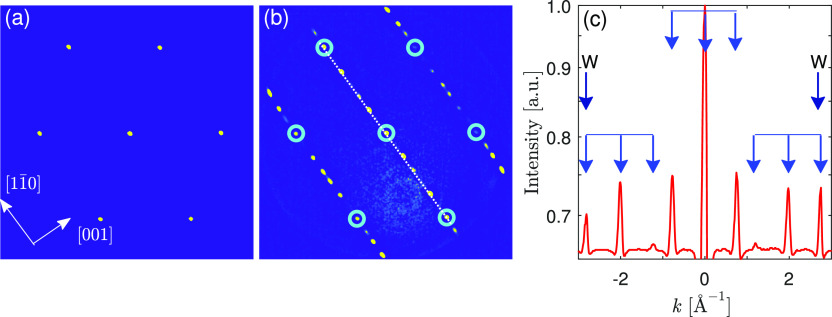
LEED patterns of (a) bare W(110) and (b) monolayer Sb on W(110). *E* = 40 eV. Circles in (b) indicate W(110) diffraction spots. The LEED patterns have been filtered with the FFT bandpass filter in order to remove the secondary electrons background. Original LEED patterns are presented in SI. (c) Intensity profile (normalized log scale) along [11̅0] of W(110) covered with Sb monolayer along the dotted line marked in (b). The arrows indicate the W and Sb induced superstructure diffraction spots.

Figure 2c presents the intensity profile taken along the white dotted line shown in Figure 2b. Besides the (00) and W-related intensity peaks, there are equally spaced additional ones associated with the new diffraction spots: all are indicated by the blue arrows. The distance of 0.77 ± 0.03 Å^–1^ between the Sb-induced and the W diffraction spots along [11̅0] indicates formation of the new periodic structure with the lattice constant of 8.2 ± 0.2 Å. The obtained periodicity agrees very well with the STM results (Figure 1a). It is important to note that the new period does not fit to the periodicity of the tungsten substrate along the [11̅0] direction. The distance between the rows of tungsten atoms along [11̅0] equals 2.24 Å and the closest multiple values are 6.72 Å (3 × 2.24 Å) and 8.96 Å (4 × 2.24 Å). It means that the Sb layer forms incommensurate structure along the W [11̅0] direction.

The large-scale growth of the Sb layer has been monitored with LEEM, as shown in Figure 3. LEEM images of the W(110) surface before (Figure 3a), during (Figure 3b), and after deposition of a full layer of Sb (Figure 3c) indicate homogeneous growth of the layer over the whole field of view, here 5 × 5 μm^2^. Thin dark lines visible in the LEEM image before Sb deposition denote monatomic steps characteristic for the bare W(110) surface. During deposition, the step contrast initially disappears, Figure 3b. It reappears when the first layer of Sb atoms becomes complete. The appearance of islands of the next layer, dark spots in Figure 3c, indicates that the first layer is full. It is important to note that the Sb atoms form a continuous and smooth film with the same morphology and crystallographic order over the sample area of several tens of mm^2^.

**Figure 3:**
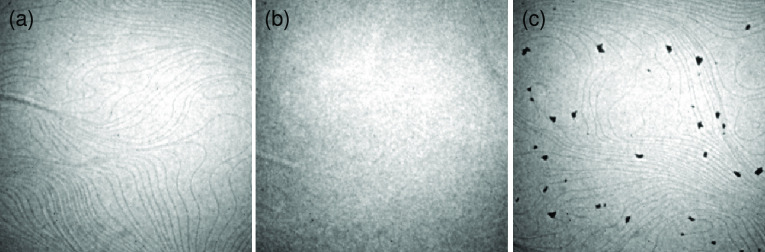
LEEM images of (a) bare W(110), (b) covered with about half of the Sb monolayer, and (c) full Sb monolayer. Field of view: 5 × 5 μm^2^. Electron energy *E* = 0.8 eV. The image shift is caused by a thermal drift (deposition temperature equals 400 K).

The crystallographic structure of the Sb layer does not resemble any known stable antimonene structures (α and β phases) or those theoretically predicted and presumed to be unstable.^1^ It is also different from the Sb structures obtained on other surfaces.^8−11,15,16^ In order to shed more light on the obtained complicated crystallographic structure of the Sb layer, DFT model calculations have been performed. Very good agreement between the DFT results and the STM measurements has been obtained for 15 Sb atoms per 1·*a*_*W*_[001]__ × 21·*a*_*W*_[11̅0]__ unit cell. Unit cells with other numbers of Sb atoms have also been tested, but with no agreement with the STM images. The corresponding phase diagram is shown in Figure S3 in the SI. The full atomic model of the Sb layer, together with the underlying W atoms, is presented in Figure 4. The arrangement of the Sb atoms repeats periodicity of the tungsten substrate along the [001] direction forming characteristic rows of atoms. According to the results of DFT calculations the Sb atoms are adsorbed on the W(110) surface without intermixing with the substrate atoms. Another distinct feature of the system is its flatness; there is no buckling of the Sb atoms.

**Figure 4:**
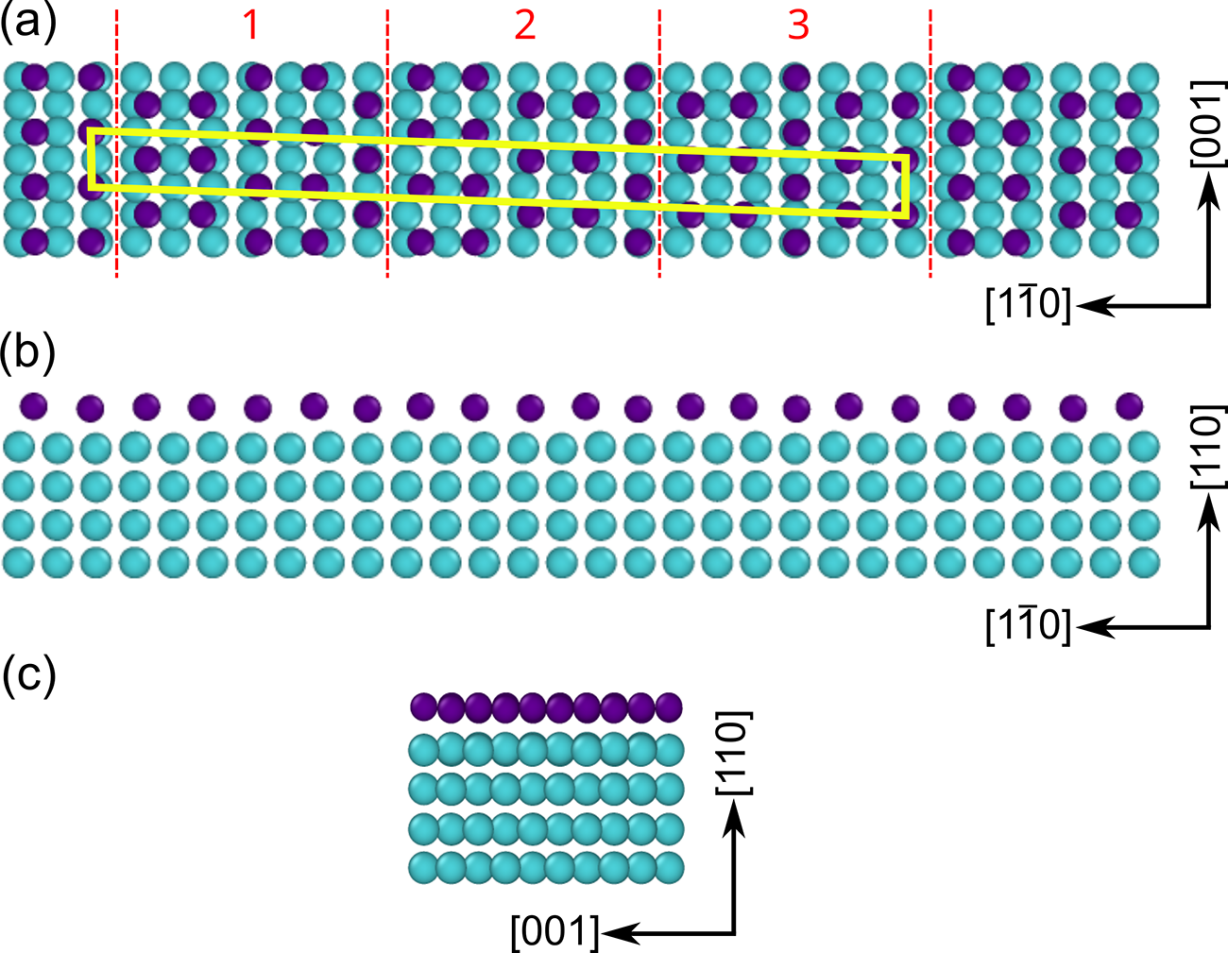
Model of Sb monolayer on W(110): (a) top and (b, c) side views. Sb (dark blue) and W (light blue) atoms. The red dashed lines in (a) separate smaller building blocks of the unit cell.

A more detailed inspection of the atomic structure reveals smaller building blocks of the unit cell, as marked by the red dashed lines in Figure 4a. There are two such blocks in the current unit cell. Third block is only slightly modified. Real surface exhibits the unit cell marked in Figure 4a, but may also contain its variations due to the third block. Obviously, this comes from the rather defected nature of the surface. Note that all corresponding structural models are degenerate, as they differ in energy by less than 10 meV per 1 × 1 unit cell.

Figure 5a shows the STM image of the Sb layer with the superimposed blue circles representing position of the Sb atoms obtained within the DFT model. Clearly, the agreement between the results of experiment and theory is very good. The simulated STM image achieved for the same sample-tip voltage as in the experiment is shown in Figure 5b. It is worth noting that relative intensity of the corresponding Sb atomic chains in both images agrees as well and additionally validates correctness of the model. Another evidence that corroborates the model is the STM image simulated at positive voltage, Figure 5c. It shows only “in-phase” Sb chains that are about 8.1 Å apart. The same distance was obtained in the STM (Figure 1a) and LEED (Figure 2b) experiments.

**Figure 5:**
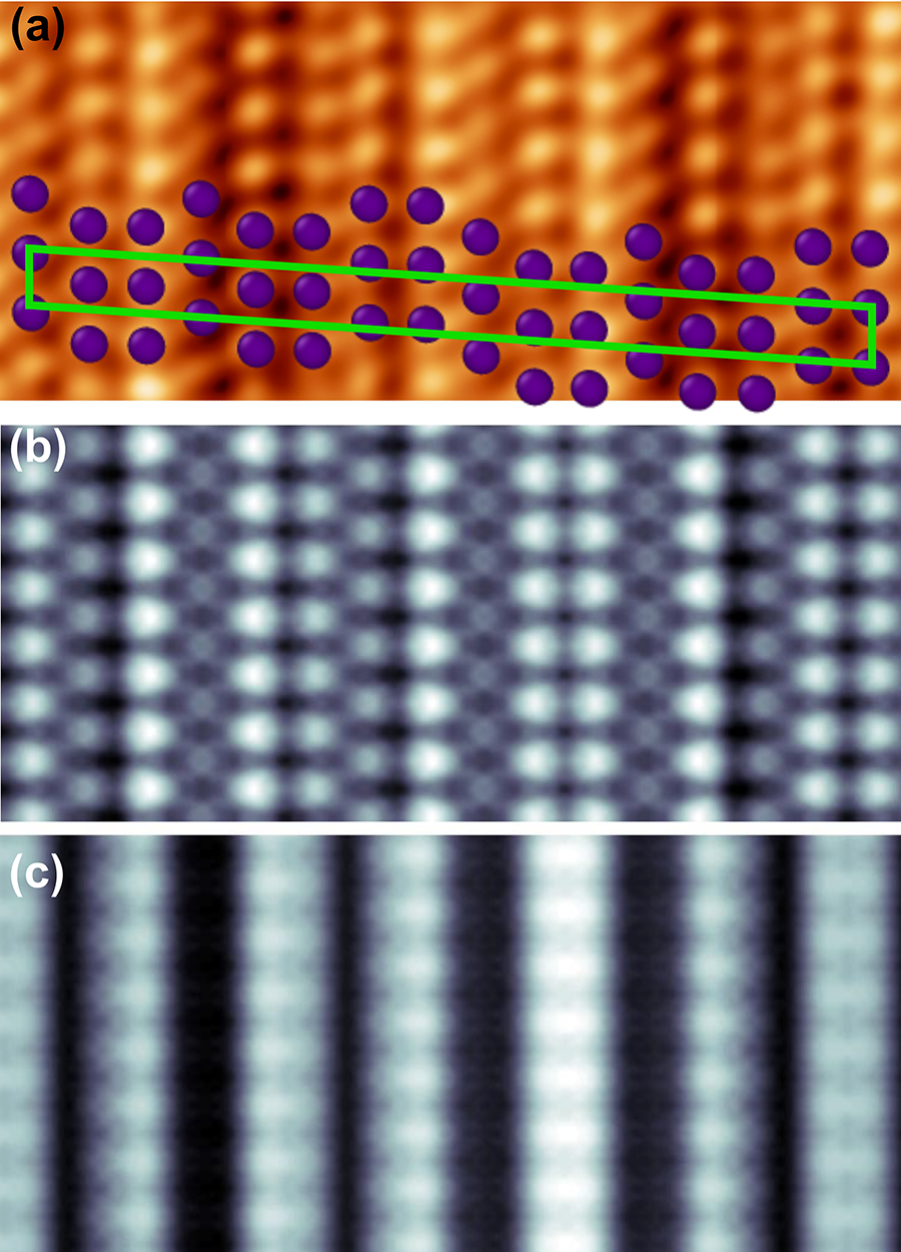
STM images (5 × 2.2 nm^2^) of Sb monolayer on W(110): (a) experiment and (b, c) simulations. *U* = −1.2 V (a, b) and *U* = +1.2 V (c), (a) *I* = 50 pA. The circles in (a) mark position of the Sb atoms according to the DFT model. Green parallelogram denotes the surface unit cell of the Sb layer.

It is interesting to note that the density of atoms of the obtained Sb layer of 10 × 10^14^ at/cm^2^ fits well to half of the atom density of the alpha phase of antimonene (according to refs (4 and 17−19), it changes from about 19.0 to 19.6 × 10^14^ at/cm^2^). Together with the fact that the layer is very flat without buckling characteristic for the alpha phase, it may suggest that it is formed by one of the sublattices of that phase of antimonene. Such a “single sublattice” of the van der Waals layers has been observed in other systems: the β phase of antimonene^20,21^ or Bi_2_Se_3_.^22^ However, those reports show relatively small crystallites or flakes that have the same or a corresponding crystallographic structure as the full layer and may be considered as the intermediate metastable forms of the full layer. Contrarily, in the current research the obtained structure is stable, fully covers macroscopic area, and obviously has different than the rectangular crystallographic structure. Therefore, it can not be considered as a part of the antimonene alpha phase.

According to our studies, the Sb atoms can be completely removed from the W(110) surface after annealing at about 800 K. The relatively high desorption temperature indicates rather strong bonds between Sb and W atoms. The results of DFT calculations give a bonding energy between W and Sb of about 1.6 eV per Sb atom. Taking into account the arrangement of the Sb atoms, which is defined by the symmetry and the position of atoms of the tungsten substrate, it can be concluded that the single-atom-thick Sb layer is stabilized by the W(110) substrate.

Summarizing, the new phase of antimonene has been prepared by molecular beam epitaxy on the W(110) surface. Scanning tunneling microscopy measurements and the results of density functional theory calculations show that the new phase is perfectly atomically flat, contrary to its van der Waals family members. The lack of buckling is caused by the significant interaction of Sb atoms with the atoms of the densely packed underlying tungsten surface. The single-atom-thick Sb film uniformly covers the area of the order of cm^2^ of the substrate as revealed by the low-energy electron microscopy. The presented findings show that besides the known forms of 2D materials, other structural phases stabilized by the substrate can also be created.

## Methods

### Experimental Section

All experiments were performed in two separate systems under ultrahigh vacuum (UHV) conditions with a base pressure in the middle of the 10^–11^ mbar range. The crystallographic structure and morphology of the antimonene layer were studied with low energy electron diffraction (LEED), scanning tunneling microscopy (STM), and low energy electron microscopy (LEEM).

W(110) was used as a substrate for the growth of antimonene. Prior to the Sb deposition, the W(110) substrate was cleaned using standard procedure: annealing at 1400 K in an oxygen atmosphere (10^–7^ mbar) and then flashing up to about 2200 K in order to remove oxygen. Sb was deposited from resistively heated effusion cell with a rate of 1 layer of antimonene per 5 min at 400 K. The single layer of Sb was prepared *in situ* in the LEEM apparatus. For the STM measurements, the Sb film was additionally covered by about 20 layers of Sb. After that, the sample was taken out from UHV and transferred to the STM system with the following mild annealing at about 500 K under UHV. It is known that the Sb layer is resistant to ambient conditions.^23^ On the other hand, annealing at about 500 K causes desorption of excess Sb from the surface, leaving only the single layer of Sb atoms that is directly attached to the tungsten substrate. Such a preparation procedure allows for the safe transfer of the sample between UHV systems, avoiding contamination of the investigated single layer of Sb atoms and the tungsten surface with agents from the air.

The LEEM images were recorded during Sb deposition at 400 K. The diffraction experiments were done at 400 K and at room temperature, resulting in the same LEED patterns. The presented LEED patterns were taken at room temperature. STM measurements were performed at 4.5 K. STM data were analyzed with the WSXM software.^24^

### Calculations

The DFT calculations have been performed using the VASP (Vienna ab initio simulation package)^25,26^ and the GGA-PBE correlation-exchange functional.^27^ A kinetic energy cutoff of 340 eV was used for the plane wave expansion of single particle wave functions. The Brillouin zone was sampled by 10 × 1 × 1 Monkhorst–Pack k-points grid.^28^ The convergence for the total energy was chosen as 10^–6^ eV between subsequent iteration steps, and the maximum force allowed on each atom during the geometry optimization was less than 0.01 eV/Å. The W(110) system has been modeled by 4 W layers. The vacuum region of 20 Å has been added to avoid the interaction between surfaces of the slab. All the atomic positions were relaxed by a conjugate gradient method, except the bottom layer. The W atoms in the bottom layer were fixed at their bulk positions. A supercell with 21 × 1 periodicity has been considered, in agreement with the experimental conditions.

## Supporting Information Available

STM image of the new antimonene phase; Raw LEED patterns of bare W(110) and the monolayer Sb on W(110); Information on other DFT models of the Sb layer (PDF)
