# Supplementary material for: Peculiar Structural Phase of a Single-Atom-Thick Layer of Antimony
Source: Nano Lett. 2023 Oct 20;23(21):9894–9. doi: 10.1021/acs.nanolett.3c02847 (PMC10636807; doi:10.1021/acs.nanolett.3c02847)
Supplement: Supplementary file 1 — nl3c02847_si_001.pdf [file nl3c02847_si_001.pdf]

Supporting Information

Peculiar Structural Phase of a Single-Atom-Thick Layer of Antimony

Agnieszka Stępniak-Dybala, Tomasz Jaroch, Mariusz Krawiec, Piotr Dróżdż, Mariusz Gołębiowski, and Ryszard Zdyb*

Institute of Physics, Maria Curie-Sklodowska University, 20-031 Lublin, Poland

E-mail: ryszard.zdyb@umcs.pl

1. STM topography

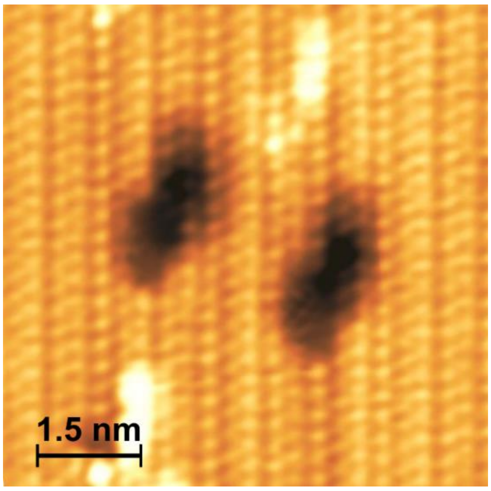

Figure S1: STM image of Sb monolayer on W(110). $U = -1.2$ V, $I = 50$ pA, 7 x 7 nm^2.

2. LEED patterns

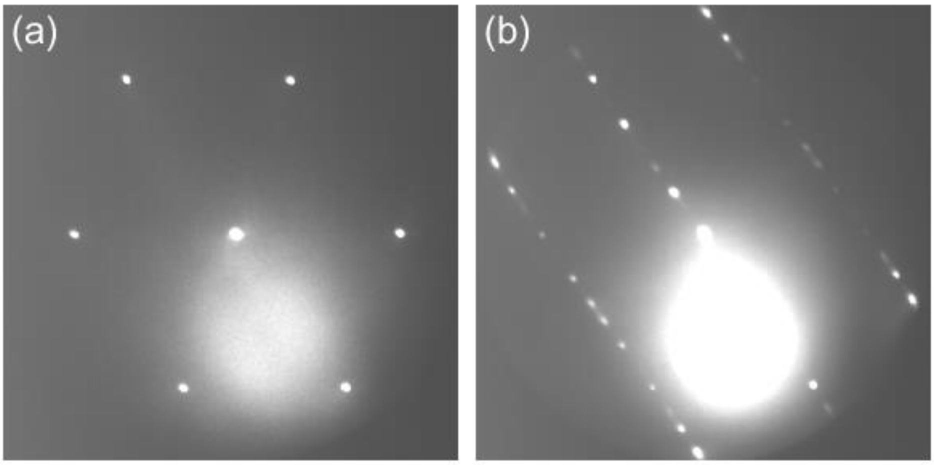

Figure S2: Raw LEED patterns of: (a) bare W(110) and (b) monolayer Sb on W(110). $E = 40$ eV. The large white spot is due to the secondary electrons.

3. DFT energetics

To elucidate the growth dynamics and the stabilities of atomic structures, we define the surface energy:

$$\gamma_{1\times1}(\mu_{Sb},\mu_W) = \frac{1}{S}[E_{slab}(N_{Sb},N_W) - E_{bare} - N_{Sb}\mu_{Sb} - N_W\mu_w],$$

where E_{slab} is the total energy of a slab containing N_{Sb} Sb atoms and N_W W atoms, and E_{bare}/S is the surface energy of the bottom bare W surface. S is the area of the 1×1 surface unit cell of W(110) bcc crystal, while μ_{Sb} and μ_W are the chemical potentials of Sb and W, respectively. In the following we define $\mu_W = E_W^{bulk}$ and $\Delta\mu_{Sb} = \mu_{Sb} - E_{Sb}^{bulk}$, where E_W^{bulk} and E_{Sb}^{bulk} are total energies of bulk W and Sb phases, respectively. Figure S3 shows the surface energies of various structures containing different number of Sb atoms. The phase diagram confirms the experimental findings that the present model (15 Sb) is the highest-density phase of the Sb monolayer.

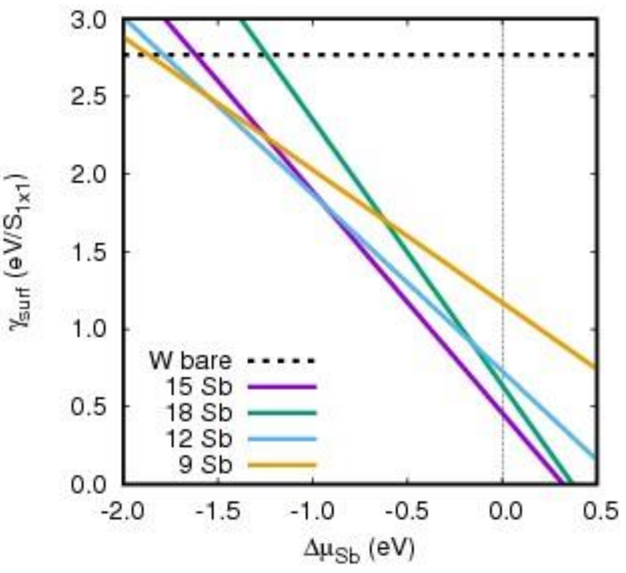

Figure S3: Surface energies of various Sb phases as a function of the Sb chemical potential.
